# Herpes simplex virus 2 and dementia risk: a systematic review and meta-analysis

**DOI:** 10.3389/frdem.2026.1737068

**Published:** 2026-03-24

**Authors:** C. J. Hunt, Brinley N. Zabriskie, Ethan J. Coulter, Morgan Chase McClellan, Grace Templeton, Reagan Erbstoesser, Scott MacLean, Chris H. Miller, Jarod Moss, Caitlyn Carter, Shawn D. Gale, Jonathan D. Moore, Thomas J. Farrer, Dawson W. Hedges

**Affiliations:** 1 Department of Biophysics, Brigham Young University, Provo, UT, United States; 2 Department of Data Analytics and Information Systems, Utah State University, Logan, UT, United States; 3 Neuroscience Center, Brigham Young University, Provo, UT, United States; 4 Department of Psychology, Brigham Young University, Provo, UT, United States; 5 Department of Biology, Brigham Young University, Provo, UT, United States; 6 Department of Psychology, California State University, Fresno, Fresno, CA, United States; 7 Department of Microbiology and Molecular Biology, Brigham Young University, Provo, UT, United States; 8 Department of Public Health, University of North Florida, Jacksonville, FL, United States; 9 School of Health and Medical Professions, University of Idaho, Moscow, ID, United States

**Keywords:** Alzheimer's disease, dementia, herpes simplex virus type 2, HSV-2, meta-analysis

## Abstract

**Introduction:**

Several potentially modifiable risk factors for dementia have been identified, including infectious diseases. Among the infectious diseases potentially associated with dementia is herpes simplex virus type-2 (HSV-2).

**Methods:**

To better characterize the association between HSV-2 and dementia, we conducted a meta-analysis of published peer-reviewed studies reporting HSV-2 exposure and dementia outcomes.

**Results:**

Of 626 identified primary studies, eight met our inclusion criteria, with one of these excluded due to overlapping data with another study, yielding seven independent studies (total *N* = 751,156). Meta-analyses found no significant association between HSV-2 infection and Alzheimer's disease (pooled odds ratios ≈ 1.1, 95% confidence intervals included the null across all methods). Similarly, when pooling odds ratios across studies examining all-cause dementia, results were non-significant (pooled odds ratios ≈ 1.2, 95% confidence intervals included 1). In contrast, pooled hazard ratios from three studies for all-cause dementia suggested a possible increased risk among individuals with HSV-2 (DerSimonian and Laird pooled hazard ratio = 1.37, 95% CI: 1.00–1.89; Hartung-Knapp-Sidik-Jonkman pooled hazard ratio = 1.35, 95% CI: 0.58–3.14), driven primarily by two significant studies.

**Discussion:**

Overall, the available evidence indicates no clear association between HSV-2 and Alzheimer's disease and only one of the two meta-analytic methods shows evidence of a potential relationship with all-cause dementia. These findings support continued investigation into the association between HSV-2 and dementia.

## 1 Introduction

By 2050, dementia cases are projected to increase from 57.4 million in 2019 to 152.8 million globally (Nichols et al., 2022), with the number of yearly dementia diagnoses in the United States expected to nearly double from 514,000 to over one million (Fang et al., 2025). With this expected rise in prevalence, increased attention has been paid to identifying risk factors for dementia and to determining the degree that such risk factors are modifiable or preventable (Prince et al., 2015). Recently, Livingston et al. (2024) identified 14 such potentially modifiable factors that, if mitigated, could reduce the global incidence of dementia by as much as 45%. While the review by Livingston et al. (2024) briefly discusses evidence of increased dementia risk associated with infectious disease, this particular risk factor was not included in their overall quantitative analysis as a variable of interest. However, an increased risk of dementia has been associated with several infectious pathogens (Bruno et al., 2023; Farrer et al., 2024; Steel and Eslick, 2015), including herpes simplex virus type-1 (HSV-1; Elhalag et al., 2023), human herpes virus-6A (HHV-6A) and human herpes virus-7 (HHV-7; Readhead et al., 2018), hepatitis C virus (HCV; Beydoun et al., 2024), human immunodeficiency virus (HIV; Lam et al., 2021), cytomegalovirus (CMV; Lee et al., 2020; Sanami et al., 2024), varicella-zoster virus (VZV; Bae et al., 2021; Marra et al., 2025), and human papillomavirus (HPV; Lin et al., 2020). Moreover, some studies have shown a decreased risk of dementia following immunization (Wu et al., 2022) or administration of various antimicrobial medications (Tzeng et al., 2018). Additionally, emerging but inconsistent evidence suggests that herpes simplex virus type-2 (HSV-2) may also negatively impact the brain and affect dementia risk (Hansen et al., 2020; Tzeng et al., 2018; Warren-Gash et al., 2019).

HSV-2 is a herpesvirus that may remain dormant in the central nervous system (CNS) of infected individuals (Berger and Houff, 2008). HSV-2 is associated with genital herpes, although many with this infection are unaware that they have it or do not recognize the symptoms [Centers for Disease Control and Prevention (CDC), 2010]. HSV-2 is generally transmitted through sexual contact, in contrast to HSV-1, which is generally transmitted through contact with saliva from an infected individual. An estimated 491.5 million people live with HSV-2 infection globally, including 18.2 million between ages 18 and 49 in the United States (Spicknall et al., 2021), with the highest percentage in women (21.9%; 102.9 million) living in the World Health Organization (WHO) African region (James et al., 2020). In 2016, the seroprevalence of HSV-2 in the United States was estimated at 11.9%, with non-Hispanic Black persons having the highest seroprevalence (34.6%) and non-Hispanic Asian persons having the lowest (3.8%; McQuillan et al., 2018).

Similar to many other infectious pathogens, HSV-2 has been associated with multiple detrimental effects on the human CNS, including decreased whole-brain cortical thickness among older adults, although not in those with an Alzheimer's disease (AD) neuroanatomical signature (Roberts et al., 2023), increased risk of ischemic stroke (Hauer et al., 2019) and seizures (Hansen et al., 2020), and increased risk of meningitis, encephalitis, and mortality (Berger and Houff, 2008). There are mixed findings about the association between HSV-2 and dementia, which may be explained, in part, by confounding or interactions related to the grouping of HSV subtypes in some studies. For example, when associations with dementia are examined in combined HSV-1 and HSV-2 samples relative to non-infected controls, there is evidence of an increased risk of AD and other dementias in the combined HSV group compared to the control group in some (Elhalag et al., 2023; Letenneur et al., 2008) but not all studies (Drinkall et al., 2025), making it difficult to understand the impact of HSV-2 specifically. When HSV-1 and HSV-2 are examined separately compared to a control group, the findings continue to be inconsistent. Hansen et al. (2020) demonstrated an increased risk of dementia associated with HSV-1 infection but not with HSV-2 infection in a study using polymerase chain reaction for the identification of HSV-2 DNA in cerebral spinal fluid. Likewise, one study meta-analyzed two primary, post-mortem brain analyses searching for HSV-2 DNA and found no increased risk of dementia (Warren-Gash et al., 2019). However, the researchers discussed an additional study reporting a strong association with an odds ratio (OR) of 4.29, [95% confidence interval (CI): 2.01–9.16] between HSV-2 in blood and vascular mild cognitive impairment, suggesting that some HSV-2 cases may progress to dementia (Deng et al., 2016; Warren-Gash et al., 2019). In another study, a population-based cohort study from Taiwan demonstrated that both HSV-1 and HSV-2 independently increased the risk of all-cause dementia compared to non-infected controls. The association when HSV-2 was examined separately, however, was weaker than the association when combined with HSV-1 (Tzeng et al., 2018). Further, Tzeng et al. (2018) found that the use of anti-herpetic medications in the treatment of HSV-1 and 2 reduced dementia risk, supporting the possible role of these pathogens in dementia risk. More recently, Beydoun et al. (2024) found that both HSV-1 and HSV-2 were independently associated with a greater risk of all-cause dementia. These mixed results indicate a need for additional research to better characterize the association between HSV-2 and dementia in diverse populations.

In this meta-analysis, we aim to build on this growing body of evidence of the role of infectious diseases in dementia by addressing inconsistencies in the literature regarding the possible association between HSV-2 infection and dementia risk. To do so, we used meta-analytic methods to examine the association between HSV-2 infection and dementia based on published primary studies.

## 2 Materials and methods

### 2.1 Study selection and data extraction

Using the Embase, ProQuest, PubMed, Scopus, and Web of Science Core Collection databases, we searched for primary studies that included reference to HSV-2 and dementia or cognitive impairment using the following Boolean structure and search terms: < “HSV 2” OR “Herpes Simplex Virus 2” OR “Herpes Simplex Virus Type 2” OR “Herpesvirus 2 (alpha), Human” OR “HHV-2” OR “HSV-2” OR “Human Herpesvirus 2”> using the broadest field permitted in each database and dementia-related terms searched in the “Title,” “Abstract,” and “Keywords” (if available) fields, including Lewy Body Dementia, Frontotemporal Dementia, Alzheimer's Disease, Mild Cognitive Impairment, Dementia and other dementia subtypes with their associated abbreviations (for the complete search, please see Supplementary materials). In short, this search strategy was designed to identify published peer-reviewed papers investigating HSV-2 and dementia subtypes or mild cognitive impairment. Our search included primary studies available before March 27, 2025.

Six independent members of the research group screened for primary studies potentially meeting inclusion criteria and then cross-checked for each full-text article using the following inclusion criteria: (1) inclusion of an HSV-2 seropositive group, (2) report of dementia outcome, (3) comparison to a healthy control group seronegative for HSV-2, and (4) living-human study. We included studies that reported 2 × 2 table data or adjusted odds, rate, or hazard ratios (Higgins et al., 2024, Ch 6.3). Initially, our inclusion criteria spanned all methods of determining HSV-2 status, such as seropositivity, post-mortem studies, and polymerase chain reaction. Because of concerns about how directly comparable these different methods were, we restricted the inclusion criteria to just samples that evaluated seropositivity.

**Figure 1:**
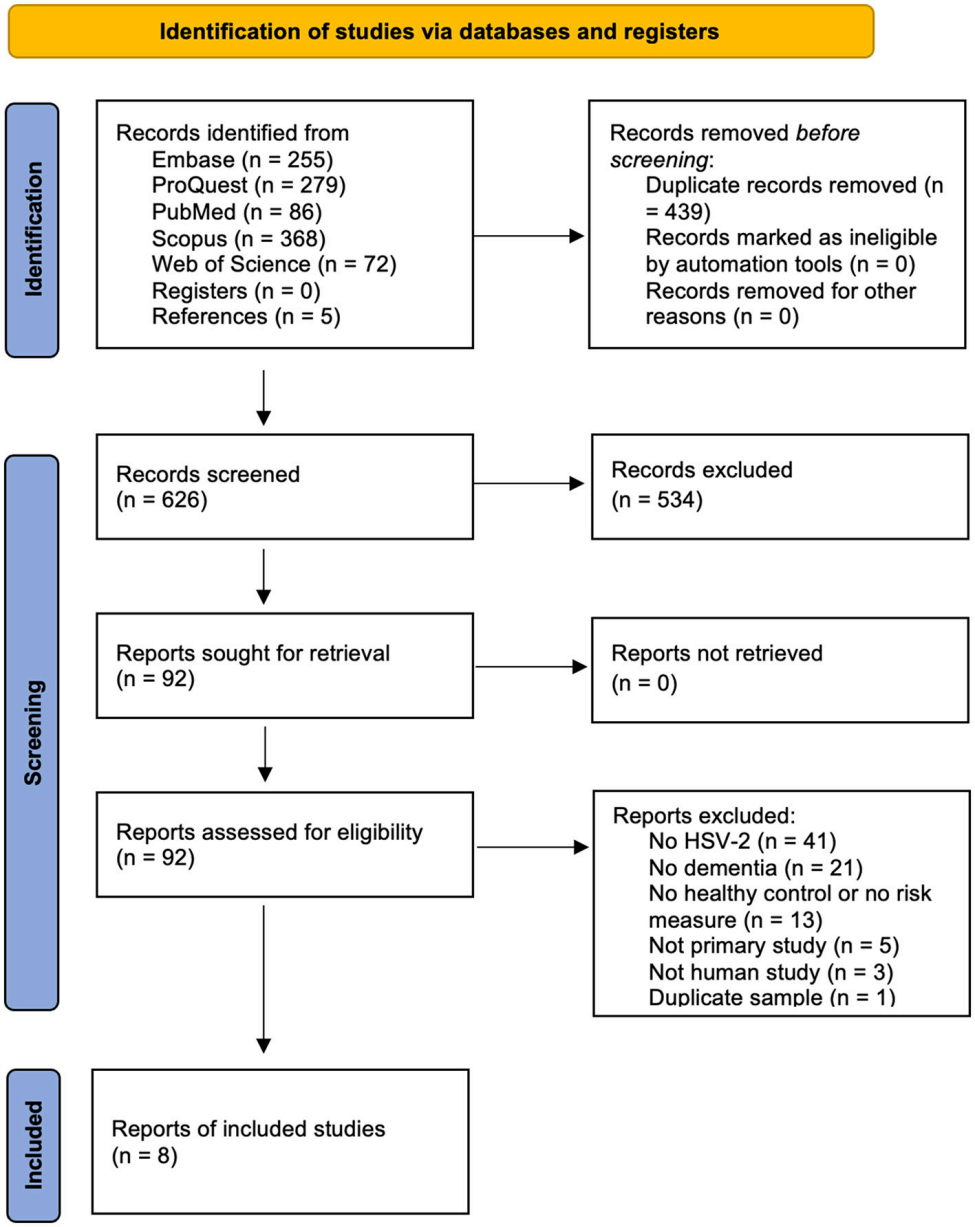
Flow chart showing the process of study identification and selection according to Preferred Reporting Items for Systematic Reviews and Meta-Analyses (PRISMA).

From identified primary studies that met inclusion criteria, we extracted when available author name, year of publication, dementia type, total sample size, number of HSV-2 positive cases, number of HSV-2 negative cases, number of dementia cases in the HSV-2 positive group, number of dementia cases in the HSV-2 negative group, mean ages, percent female, socioeconomic status of sample, racial/ethnic background of sample, location of study, and variables used for adjustment of effect measures. Any discrepancies were resolved by consensus with other team members. Additional details of this study selection and data extraction process are shown in Figure 1 and reported according to Preferred Reporting Items for Systematic Reviews and Meta-Analyses (PRISMA) standards (Page et al., 2021).

### 2.2 Risk of bias assessment for included studies

To evaluate risk of bias in the included primary studies, we used the Newcastle-Ottawa Scale (NOS), a risk of bias assessment designed for non-randomized cohort and case-control studies used in meta-analyses (Wells et al., 2000). The NOS consists of a series of questions across three domains—the selection of cases and controls, the control of important confounding variables, and the ascertainment of exposure for case control studies or assessment of outcome for cohort studies, with a higher total score indicating a lower risk of bias (Wells et al., 2000). Four independent coders completed the NOS for each primary study included in the meta-analysis using the scale provided for case/control and cohort studies. Any discrepancies were resolved by consensus with other members of the study group.

### 2.3 Statistical analysis

We planned four primary meta-analyses based on the included studies. Two focused on the association between HSV-2 and all-cause dementia—one combining studies reporting odds ratios (ORs) and the other combining those reporting hazard ratios (HRs) from Cox proportional hazard models. The remaining two meta-analyses examined HSV-2 in relation to AD, again with separate analyses for ORs and HRs. We obtained unadjusted log odds ratios (logORs) from 2 × 2 table data, and when needed, we transformed reported ORs and HRs to logORs and log hazard ratios (logHRs). We also obtained associated measures of precision. In line with the *Cochrane Handbook for Systematic Reviews of Interventions* (Higgins et al., 2024, Ch 24.4.1), which recommends prioritizing adjusted over unadjusted estimates, our analyses used adjusted ORs when available, while including unadjusted estimates when no adjusted values were reported. As a sensitivity analysis, we compared results obtained using unadjusted 2 × 2 table estimates to assess the consistency of our findings.

Although both OR and HR effect measures can quantify associations between HSV-2 and dementia, they capture different aspects of risk. ORs are based on binary outcomes measured at a fixed time point, whereas HRs arise from time-to-event analyses and incorporate information on the timing of dementia onset as well as censoring. As a result, the two effect measures are not directly comparable, as ORs and HRs quantify fundamentally different aspects of risk. To ensure appropriate synthesis and interpretation, we therefore analyzed ORs and HRs separately.

For all meta-analyses, we planned to use random-effects (RE) models given the expected heterogeneity in baseline sample characteristics, diagnostic methods, and HSV-2 diagnoses (Bender et al., 2018; Schulz et al., 2022). Additionally, following guidance from the *Cochrane Handbook for Systematic Reviews of Interventions*, we applied multiple meta-analysis models, as recommended when synthesizing results from only two or three studies, given the increased methodological uncertainty associated with small-sample meta-analyses (Higgins et al., 2024, Ch 10.10.4.5).

#### 2.3.1 Meta-analysis of studies with odds ratios

We conducted two separate meta-analyses based on distinct sets of studies that reported 2 × 2 table data and met our inclusion criteria: one analyzing the association between HSV-2 and all-cause dementia (three studies), and the other examining HSV-2 and AD (a different set of three studies, with one study appearing in both analyses). From these data, we computed logORs as the effect sizes for each study, along with their associated standard errors.

Conducting meta-analyses involving only a few primary studies presents several challenges. If the heterogeneity variance (τ^2^) is large, pooling effect sizes should not be performed (Bender et al., 2018). However, the estimators used to assess heterogeneity variance often fail to produce reliable estimates when the number of studies is small (Higgins et al., 2009). To help address this, we applied several heterogeneity variance estimators, Cochran's *Q*-test for heterogeneity (Sutton et al., 2000), a visual inspection of the forest plot, and a 95% prediction interval to better understand the heterogeneity variance. A prediction interval estimates the range where the true effect of a new study from the same population is expected to fall, and its use has been recommended to better understand between-study differences (Higgins et al., 2009; Veroniki et al., 2019). However, with so few studies, the prediction intervals are expected to be very wide and therefore largely uninformative, reflecting the uncertainty in our estimates (Borenstein, 2023).

If the heterogeneity variance is not excessive, we can proceed with an RE model. However, with few primary studies, the widely used DerSimonian and Laird (DL) RE method typically produces overly narrow confidence intervals (CIs), as noted by Felsch et al. (2022) and Cornell et al. (2014). The DL method estimates the heterogeneity variance using a method-of-moments estimator derived from Cochran's *Q*-test, and constructs CIs assuming a normal distribution. Importantly, it does not account for the uncertainty in estimating the heterogeneity variance, which can be substantial when the number of studies is small. Since τ^2^ is difficult to estimate with limited data, failing to incorporate this uncertainty can lead to CIs that are too narrow, increasing the risk of a Type I error.

The Hartung–Knapp–Sidik–Jonkman (HKSJ) method (Hartung, 1999; Knapp and Hartung, 2003; Sidik and Jonkman, 2002) has been used as an alternative in these scenarios. This method adjusts the standard error of the pooled estimate and uses a *t*-distribution with k-1 degrees of freedom (here, *k* = 3, the number of studies for both meta-analyses) when creating CIs. Unlike the DL method, which treats the estimated heterogeneity variance as fixed, the HKSJ method incorporates the uncertainty in estimating τ^2^. To estimate the heterogeneity variance with the HKSJ method, the Paule–Mandel estimator has been recommended (Mathes and Kuss, 2018).

Despite its advantages, the HKSJ method can overestimate the uncertainty in estimating τ^2^, producing CIs that are too wide or non-informative when applied to meta-analyses with few studies (Felsch et al., 2022). Current recommendations suggest comparing the HKSJ results with those from the DL method (Schulz et al., 2022). If the HKSJ CIs are narrower than those produced by the DL method, which may occur when heterogeneity is low, an *ad-hoc* variance correction is recommended (Schulz et al., 2022).

Given the limitations of the HKSJ and DL methods in meta-analyses with few studies, alternative methods have been explored. One such approach is the beta-binomial model, which, although used less frequently than the traditional RE model, has been proposed as a useful alternative when the number of studies is small (Felsch et al., 2022; Mathes and Kuss, 2018). Unlike the RE model, which estimates study-specific effect sizes and their variability, the beta-binomial model directly models the event counts. As neither method is universally successful for meta-analyses with few studies, it is often recommended to use the beta-binomial model alongside a RE model for a more comprehensive analysis (Felsch et al., 2022).

In this study, we applied the standard “common-rho” frequentist beta-binomial model, constructing CIs using a *t*-distribution with 2k-2 degrees of freedom. This model assumes that the probability of an event (e.g., dementia) in each group (HSV-2 or control) varies across studies, reflecting differences in study populations or settings. These probabilities are modeled using a beta distribution, which captures both the average event rate and its variability. By incorporating this variability, the beta-binomial model naturally accounts for heterogeneity between studies.

Low event rates introduce additional methodological challenges. When relatively few individuals in the included studies are diagnosed with all-cause dementia or AD, the information available for estimating effect sizes becomes even more limited. This scarcity of events, coupled with a small number of studies, exacerbates the difficulty of obtaining reliable meta-analytic estimates. To address this, we applied the previously described methods that have been recommended for settings with few studies. Additionally, we incorporated a method shown to be a top performer with rare event data: the Mantel-Haenszel (MH) RE model combined with the Improved Paule–Mandel (IPM) estimator of the heterogeneity variance (Bhaumik et al., 2012; Mantel and Haenszel, 1959; Zabriskie et al., 2023).

#### 2.3.2 Meta-analysis of studies with hazard ratios

As with the OR data, we planned to perform two separate meta-analyses based on studies that reported HRs from Cox proportional hazards models: one examining the association between HSV-2 and all-cause dementia, and the other between HSV-2 and AD. Some studies did not report a standard error directly, so we calculated the standard error from the reported 95% confidence intervals.

Given the differences in the studies' underlying Cox model specifications, time-to-event definitions, and covariate adjustments, we anticipated some degree of heterogeneity. We formally assessed heterogeneity using Cochran's *Q*-test and estimates of the heterogeneity variance. We followed similar methodological recommendations as in the OR meta-analyses, applying both the HKSJ and DL RE methods. The beta-binomial approach was not applicable here, as it requires event counts that are only available from 2 × 2 table data.

#### 2.3.3 General considerations for the meta-analyses

When working with a limited number of studies—especially those involving rare events—statistical methods may fail to detect an effect, even when its presence is strongly suggested based on the study-level data and the research question. In such cases, it may still be possible to infer evidence of a “conclusive effect,” where an effect can be identified directionally (protective vs. damaging), but estimating its exact size is not feasible or meaningful [Institute for Quality Efficiency in Health Care (IQWiG), 2023]. The Institute for Quality and Efficiency in Healthcare (IQWiG) outlines when this approach is appropriate in their General Methods handbook (Institute for Quality Efficiency in Health Care (IQWiG), 2023):

1. The prediction interval does not cover the null value, or
2. If the null value is included, but the effect estimates of at least two studies are conclusive, the following criteria must be met for those studies:
  a. The total weight (derived from a RE model) of the studies with conclusive results is 80% or greater,
  b. At least two studies suggest statistically significant results, and
  c. At least 50% of the weight of these studies is based on statistically significant results.

We evaluated whether our meta-analyses met these conditions for drawing a conclusive effect.

Due to the small number of studies included, we did not perform meta-regression or publication bias analyses, as these methods require a larger number of studies to produce meaningful results (Higgins et al., 2024, Ch. 10.11.4 and 13.3.4.4).

Lastly, we considered removing studies with a high risk of bias according to the NOS as *post-hoc* sensitivity analyses (Higgins et al., 2024, Ch. 10.10.3).

### 2.4 Software

All analyses were performed using the R Statistical Software (v4.4.2; R Core Team, 2024). We used the *metafor* package for the HKSJ and DL RE models (Viechtbauer, 2010). The beta-binomial model was implemented using code provided by Jansen and Holling (2023) in their study on Bayesian approaches to rare event meta-analysis (Jansen, 2023). For the MH RE model with the Improved Paule–Mandel (IPM) estimator of heterogeneity variance, we used the *meta* package (Balduzzi et al., 2019), incorporating an R script we developed to obtain the IPM estimate.

## 3 Results

Our literature search identified 626 unique studies, of which eight met our inclusion criteria (Araya et al., 2025; Beydoun et al., 2024; Fu et al., 2024; Lindman et al., 2021; Liu et al., 2025; Lövheim et al., 2018; Mekli et al., 2022; Tzeng et al., 2018; see Figure 1). Lindman et al. (2021) and Lövheim et al. (2018) appeared to report data on overlapping populations, so we decided to include only Lövheim et al. (2018) because it reports a greater sample size, bringing the total number of included studies to seven, for a total of 751,156 participants (Table 1). Based on the NOS, no studies were classified as high risk of bias, all achieving a score of 7 out of 9 or higher.

**Table 1:** Characteristics of included studies.

**Study details**		**Total sample**				**+HSV-2**	**–HSV-2**	**HSV-2 diagnosis**			**Dementia diagnosis**		
**Author, year**	**Geographic location**	**Total sample**	**Mean age, years**	**% Female sample**	**Covariates**	**Cases**, ***n***	**Control**, ***n***	**Diagnosis method**	**Who made diagnosis**	**Antiviral treatment**	**Diagnosis method**	**Who made diagnosis**	**Cognitive tests and scales**
Araya et al. (2025)	United States	6,505	All over 65 years old	–	Age at HSV test, sex, race, ethnicity, and comorbid healthcare outcomes	3,218	3,287	Any instance of a positive HSV2 pathogen test using LOINC codes	–	–	ICD-10 codes		–
Beydoun et al. (2024) ^a^	United States	2,974	59.5 ± 0.5	54.8 ± 0.3%	Age, sex, race, income, education, urbanicity, household, marital status, diet quality and biomarkers, lifestyle and health, dental status, and social support	24.9%	75.1%	Positive reaction to gG-2 antigen by immunoblot	NHANES database	–	ICD-9 codes	Record from inpatient, skilled nurse practitioner, home health agency, health options program, or carrier claims	ICD-9 diagnostic code linkage
Fu et al. (2024)	United Kingdom	8,144	56.47	56.3	Age, sex, education, APOE4, and date of entry in the queue	1,313	6,831	IgG antibody titers with median fluorescence intensity. Multiplex serological testing	UK Biobank suggested cut-offs	–	ICD-9 and ICD-10 codes	Hospital, primary care, and mortality records	ICD diagnostic code linkage
Lindman et al. (2021)	Sweden	678	61.3 ± 5.6	76.4	Matched by age, sex, sampling date, cohort	92	586	ELISA using anti-HSV IgG then anti-HSV-2 IgG	–	–	DSM4 and Neuroimaging	Memory clinic at the Umea University Hospital	–
Liu et al. (2025)	United States	689,256	73.38 ± 5.48	65.11	Age, sex, region, database entry year, and inpatient/outpatient visit numbers	959	688,297	ICD-9 054.1x, ICD-10 A60.x	–	Antiherpetics reported	ICD-9 and ICD-10 codes	–	–
Lövheim et al. (2018)	Sweden	720	61.2 ± 5.6	75.3	Age, sex, sampling date, cohort	98	622	ELISA using anti-HSV IgG then anti-HSV-2 IgG	According to manufacturer's recommendations	–	DSM4 and Neuroimaging	Psychogeriatric medicine specialist	–
Mekli et al. (2022)	United Kingdom	9,431	66.9	55.96	Age at last follow up or dementia diagnosis date	1,525	7,906	ELISA using HSV-2 IgG	–	–	ICD-9 and ICD-10 codes	–	–
Tzeng et al. (2018)	Taiwan	33,448	30.8% between 50 and 64; 69.2% over 65	43.51	Age group, sex, income, geographical area and urbanization of residence, hospital levels, comorbidity (CCI), and index year	–	25,086	ICD-9-CM codes for genital herpes 054.1, confirmed with positive ELISA HSV-2 IgG antibody or PCR	Dermatologists or infection specialists, licensed medical records technicians, and various specialists	Antiherpetics reported	ICD-9 codes	Board-certified psychiatrists or neurologists	ICD-9 diagnostic code linkage

^a^Beydoun et al. (2024) values for mean age, % female, education, and seroprevalence calculated as weighted means ± standard error, reflecting the survey complexity of the National Health and Nutrition Examination Survey (NHANES) dataset.

DSM is the diagnostic and statistical manual of mental disorders. ICD is the international classification of diseases.

### 3.1 Meta-analysis of studies assessing the association between HSV-2 and AD

Two studies reported HRs examining the relationship between HSV-2 and AD (Beydoun et al., 2024; Tzeng et al., 2018). However, one of them (Tzeng et al., 2018) reported a logHR of zero without a corresponding measure of precision. As a result, we were unable to meta-analyze those two studies and instead discuss them qualitatively in the Discussion section. This reduces the number of primary meta-analyses we conducted from four to three.

Three additional studies (Fu et al., 2024; Liu et al., 2025; Lövheim et al., 2018) reported 2 × 2 table data, allowing us to compute logORs and associated standard errors summarizing the association between HSV-2 and AD. Lövheim et al. (2018) reported an adjusted OR in addition to 2 × 2 table data. Notable differences in demographics, covariates, and diagnoses across these studies are reported in Table 1.

#### 3.1.1 Odds ratio results

This analysis combined unadjusted ORs derived from 2 × 2 table data reported in Liu et al. (2025) and Fu et al. (2024), together with an adjusted OR from Lövheim et al. (2018), obtained from a logistic regression model controlling for age, sex, cohort, and sampling date through participant matching. Cochran's *Q*-test indicated heterogeneity among the included studies (*p* = 0.04), with estimates of τ^2^ ranging from 0.22 to 0.24. While this suggests some variability, the level of heterogeneity remained within a range that allows for quantitative aggregation of the effect sizes.

**Figure 2:**
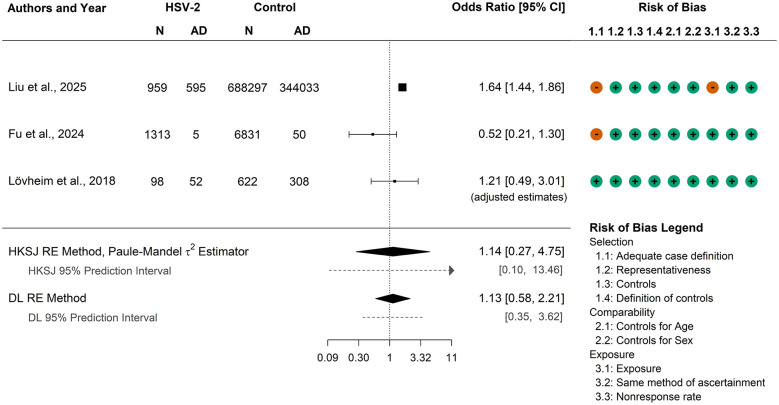
Meta-analysis results on the association between HSV-2 and Alzheimer's disease combining two studies with unadjusted odds ratios from 2 × 2 table data (Liu et al., 2025; Fu et al., 2024) with one study that reported an adjusted odds ratio from logistic regression (Lövheim et al., 2018). Two meta-analysis methods were applied to provide a more comprehensive analysis, and 95% prediction intervals are also reported. HSV-2, herpes-simplex virus 2; CI, confidence interval; N, sample size; AD, Alzheimer's disease; HKSJ, Hartung–Knapp–Sidik–Jonkman; RE, random-effects; DL, DerSimonian–Laird.

Both the typically anticonservative DL (*OR* = 1.13, 95% CI: 0.58, 2.21) and the potentially overconservative HKSJ (*OR* = 1.14, 95% CI: 0.27, 4.75) methods produced CIs that include the null value, suggesting no significant association between HSV-2 and AD, as seen in Figure 2. The 95% prediction intervals also included the null value, and only one study (Liu et al., 2025) showed a significant association, making it inappropriate to conclude a conclusive effect. Note that the beta-binomial model could not be applied as it requires adjusted 2 × 2 table counts rather than precomputed effect sizes such as those reported by Lövheim et al. (2018).

To examine the impact of using the adjusted effect from Lövheim et al. (2018), we repeated the analysis with the study's unadjusted OR. The unadjusted estimate of the association between HSV-2 and AD in this study similarly included the null value (*OR* = 1.15, 95% CI: 0.75, 1.77). Heterogeneity was again present (Cochran's *Q*-test *p*-value = 0.02), with τ^2^ estimates ranging from 0.05 to 0.22 (DL estimate = 0.13, Paule–Mandel estimate = 0.22). As with the adjusted analysis, both the DL (*OR* = 1.18, 95% CI: 0.72, 1.92) and HKSJ (*OR* = 1.13, 95% CI: 0.30, 4.24) methods indicated no significant association. In this case, the beta-binomial model could be applied, and it likewise produced a non-significant result (*OR* = 0.99, 95% CI: 0.16, 6.28). Thus, whether using the adjusted or unadjusted estimate from Lövheim et al. (2018), there is no evidence for a conclusive association between HSV-2 and AD.

### 3.2 Meta-analyses of studies assessing the association between HSV-2 and all-cause dementia

For the association between HSV-2 and all-cause dementia, three studies (Araya et al., 2025; Fu et al., 2024; Mekli et al., 2022) provided 2 × 2 table data, from which we obtain logORs and standard errors. In Araya et al. (2025), the 2 × 2 data reflect propensity score-matched cohorts, so the corresponding OR is already adjusted for sex, ethnicity, race, several dementia risk factors (including stroke, traumatic brain injury, hypertension, depression, diabetes, etc.), and age at positive HSV-2 test. In addition to unadjusted 2 × 2 table data, Mekli et al. (2022) also reported adjusted ORs from a logistic regression model controlling for age as greater or less than 65 years old (see Table 1).

Araya et al. (2025), along with two additional studies, Beydoun et al. (2024); Tzeng et al. (2018) reported HRs from Cox proportional hazard models on the association between HSV-2 and all-cause dementia. These studies differed in their study design and analysis. Beydoun et al. (2024) used the United States' National Health and Nutrition Examination Survey (NHANES) with primary care linkage for a follow-up period of up to 30 years, with time-to-event calculated as the age, greater than or equal to 45 years at the baseline visit, until death, censoring, or dementia incidence. On the other hand, Tzeng et al. (2018), using Taiwan's National Health Insurance Research Database (NHIRD), analyzed a follow-up period of 10 years, excluding individuals younger than 50 years old or with diagnosed HSV-2 or dementia before the start of the study. In their analysis, Tzeng et al. (2018) provides HRs for all dementia cases, excluding dementia in the first year of follow-up, and excluding dementia in the first 5 years of follow-up. For our analysis, we chose to use the data for excluding dementia in the first year of follow-up. Dementia generally has a slow onset, with prodromal symptoms believed to begin potentially decades prior to an individual getting diagnosed with dementia (Lespinasse et al., 2023). Those who developed dementia during the first year of follow-up likely had the beginnings of dementia during the start of this study. Additionally, in an effort to reduce heterogeneity, we used the data that excluded dementia in the first year of follow-up to approximate the methods used by Araya et al. (2025), who excluded patients who received a dementia diagnosis less than 1 year after they were infected. Covariates included in each paper's analysis are summarized in Table 1, along with other demographic and design differences.

#### 3.2.1 Odds ratio results

This analysis combined the unadjusted OR from Fu et al. (2024) with adjusted ORs from Mekli et al. (2022) and Araya et al. (2025). Cochran's *Q*-test indicated heterogeneity among the studies (*p* = 0.002), with estimates of the heterogeneity variance ranging from 0.17 to 0.33 (DL estimate = 0.33, Paule–Mandel estimate = 0.26). While these results point to variability across studies, the degree of heterogeneity was not sufficient to preclude pooling effect sizes.

**Figure 3:**
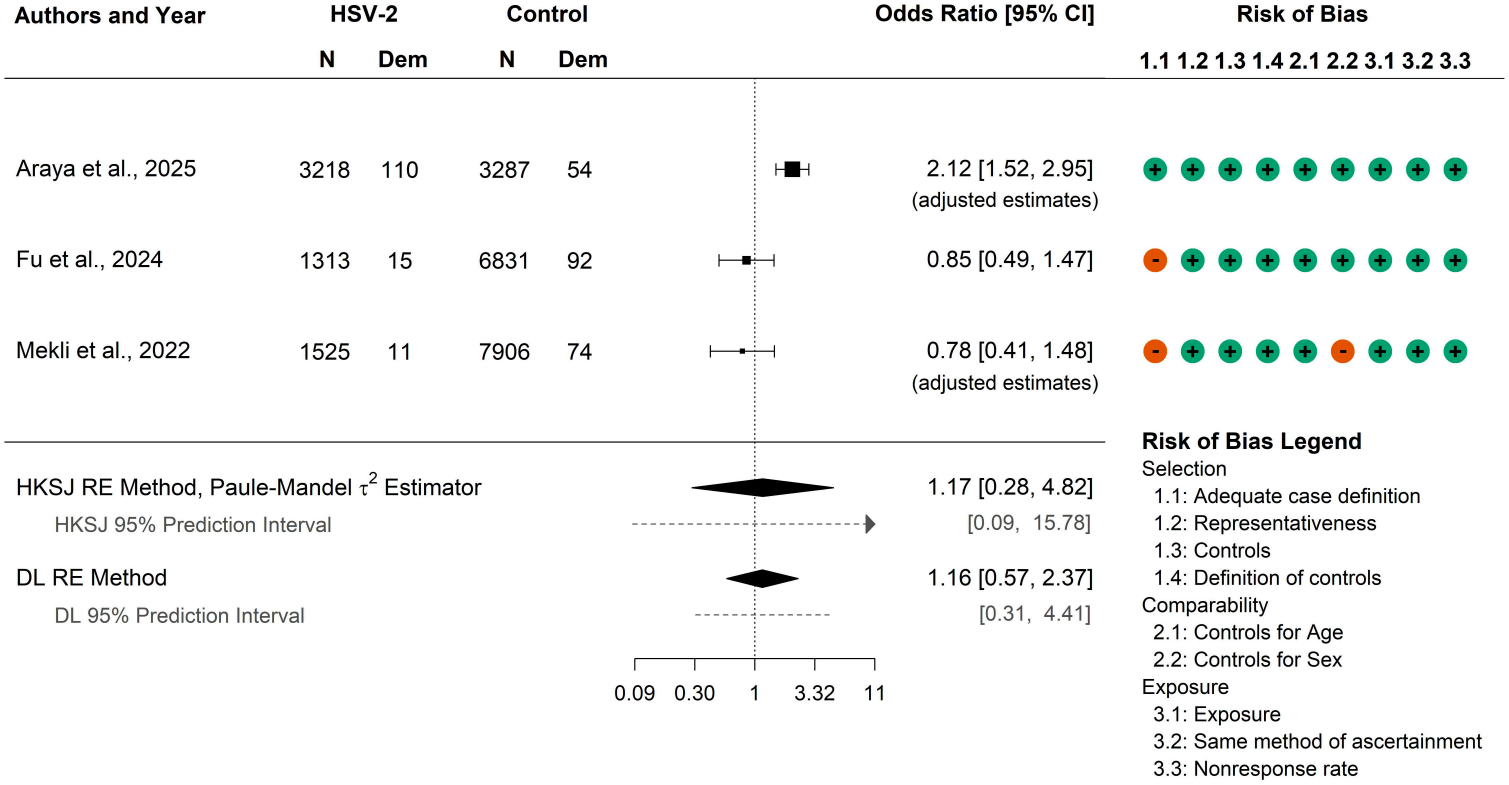
Meta-analysis results on the association between HSV-2 and all-cause dementia combining one study with an unadjusted odds ratio from 2 × 2 table data (Fu et al., 2024), with two studies reporting adjusted odds ratios (Araya et al., 2025; Mekli et al., 2022). Two meta-analysis methods were applied to provide a more comprehensive analysis, and 95% prediction intervals are also reported. HSV-2, herpes-simplex virus 2; CI, confidence interval; N, sample size; Dem, dementia; HKSJ, Hartung–Knapp–Sidik–Jonkman; RE, random-effects; DL, DerSimonian–Laird; MH, Mantel–Haenszel; IPM, improved Paule–Mandel.

The DL (*OR* = 1.16, 95% CI: 0.57, 2.37) and HKSJ (*OR* = 1.17, 95% CI: 0.28, 4.82) methods both produced CIs that cover the null value, suggesting no significant association between HSV-2 and all-cause dementia (Figure 3). The 95% prediction intervals also covered the null value, and there is no evidence of a conclusive effect.

Event rates from the 2 × 2 table data were uniformly low: in the HSV-2 arm they ranged from 0.7% to 3.4%, and in the control arm from 0.9% to 1.6%. Given this rarity of outcomes, we conducted a second meta-analysis using the unadjusted estimate from Mekli et al. (2022), which enabled application of the MH RE model with the IPM estimator of heterogeneity variance, as recommended for rare event data. The availability of full 2 × 2 table data allowed us to apply the beta-binomial model, as well.

Results from this analysis aligned closely with those of the adjusted analysis. All methods produced CIs that clearly include the null value (DL *OR* = 1.15, 95% CI: 0.56, 2.37; HKSJ *OR* = 1.16, 95% CI: 0.28, 4.85; MH *OR* = 1.17, 95% CI: 0.63, 2.18; Beta-Binomial *OR*: 1.16, 95% CI: 0.53, 2.53). Overall, these findings suggest no significant difference in the occurrence of all-cause dementia between individuals with HSV-2 and those without, regardless of whether adjusted or unadjusted estimates were used.

#### 3.2.2 Hazard ratio results

Lastly, we conducted a meta-analysis of the three studies with logHRs for all-cause dementia (Araya et al., 2025; Beydoun et al., 2024; Tzeng et al., 2018). Estimates of the heterogeneity variance ranged from < 0.01 to 0.09, with Cochran's *Q*-test yielding a *p*-value of 0.12. The DL estimate was 0.04, and the Paule–Mandel estimate was 0.07, suggesting minimal heterogeneity. Nonetheless, in line with our pre-specified analysis plan, we report random-effects results.

**Figure 4:**
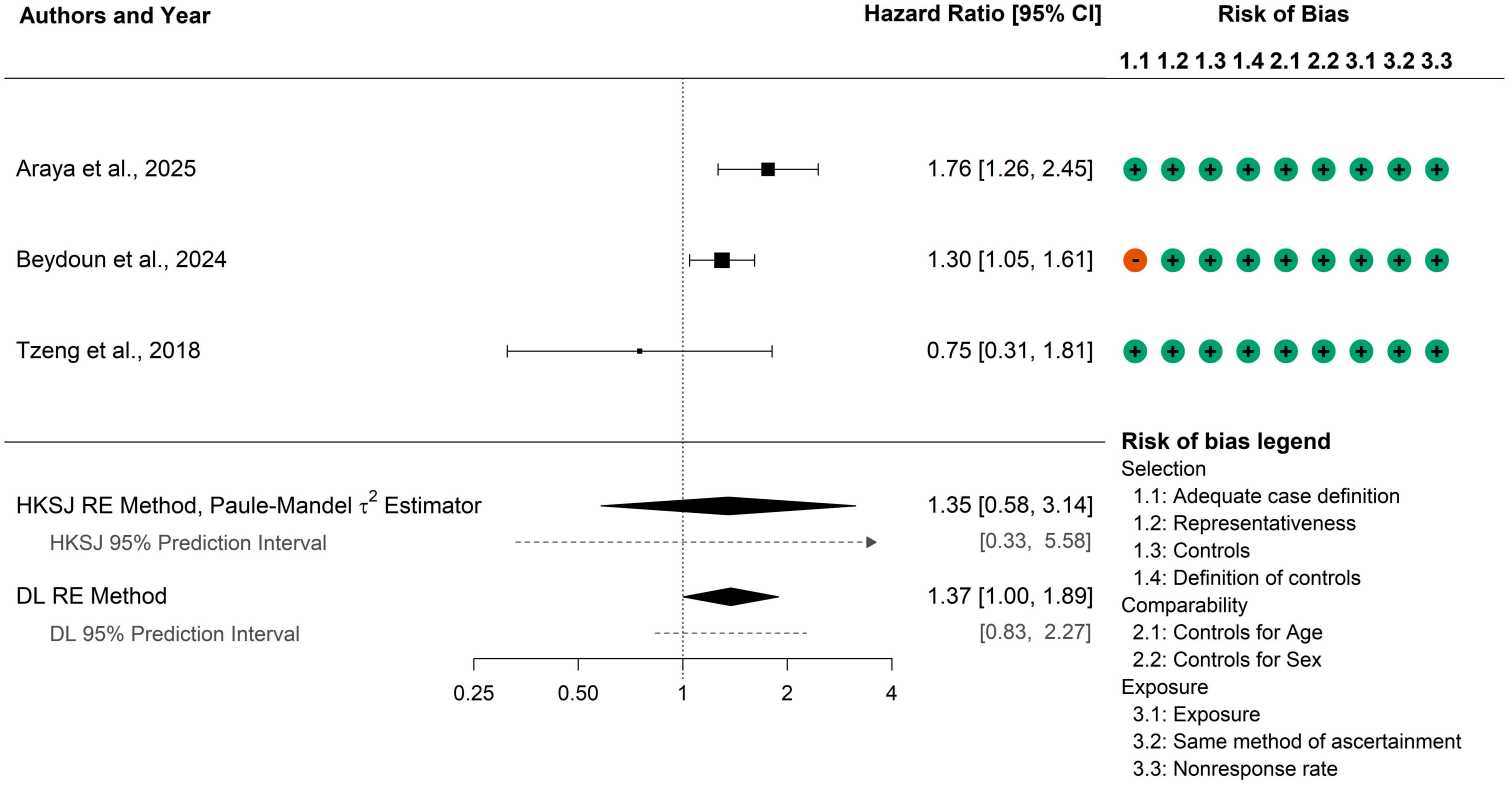
Meta-analysis results on the association between HSV-2 on all-cause dementia using studies reporting hazard ratios. CI, confidence interval; HKSJ, Hartung–Knapp–Sidik–Jonkman; DL, DerSimonian–Laird; RE, random-effects.

Using the DL method, the pooled HR was 1.37 (95% CI: 1.00, 1.89), while the HKSJ method produced a nearly identical point estimate (*HR* = 1.35) but with a much wider 95% CI (0.58 to 3.14, see Figure 4). The contrast between these results illustrates the challenges of synthesizing evidence from only three studies: the DL method may give overly narrow intervals, whereas the HKSJ method incorporates uncertainty but can yield overly wide CIs. While both 95% prediction intervals included the null value, the results still indicate a conclusive effect under the IQWiG criteria. Specifically, two studies (Araya et al., 2025; Beydoun et al., 2024) were individually statistically significant and together accounted for 86% of the study weight, thereby meeting the thresholds outlined for establishing a conclusive directional effect. Thus, while the exact magnitude of the association cannot be estimated with confidence given the limited number of studies, the evidence suggests that HSV-2 infection is associated with an increased risk of all-cause dementia.

## 4 Discussion

Increasing evidence implicates infectious agents as possible risk factors of dementia (Pinchera et al., 2024), and in this meta-analysis, we sought to better characterize the association between HSV-2 and dementia. Eight primary studies met our inclusion criteria, from which we included seven in the analyses.

Although the results of the meta-analyses of the primary studies using ORs did not show evidence of an association between HSV-2 and either AD or all-cause dementia, the results of the HR meta-analysis indicate a possible association between HSV-2 and all-cause dementia. While the DL pooled HR in this analysis only approaches significance (*HR*: 1.37, 95% CI: 1.00–1.89) and the HKSJ confidence interval is 0.58 to 3.14, a conclusive effect can be drawn from the included primary studies according to IQWiG criteria. Given that two of the three included primary studies (Araya et al., 2025; Beydoun et al., 2024) contributed to at least 80% of the overall weight of the meta-analysis, and that they each showed a positive association between HSV-2 and dementia, we can generally conclude that the HR between HSV-2 and dementia is significantly above 1, though the exact magnitude is still unknown. However, in that only three primary studies were included in this meta-analysis, that the DL pooled HR was only marginally significant, and that the HKSJ had a wide confidence interval, caution is required in interpreting these findings, as they indicate only a possible association between HSV-2 and all-cause dementia.

Additionally, we were unable to perform our planned meta-analysis looking at HSV-2 and AD using HRs due to one of the two studies reporting a logHR of zero with no corresponding measure of precision. Although not included in our meta-analysis, these two studies merit discussion here as neither found an association between HSV-2 and AD. In one, Tzeng et al. (2018) used the NHIRD from Taiwan to investigate the association between HSV-2 and AD. Using multivariate Cox proportional hazards regression analyses with covariates looking at sex, age group, geographic residence, urbanization, type of hospital, income, and a comorbidity index, Tzeng et al. (2018) found an adjusted HR (aHR) of 0 (*p* = 0.961) when looking at HSV-2 and AD for all AD cases, an aHR of 0 (*p* = 0.980) when excluding AD diagnoses in the first year of the study, and an aHR of 0 (*p* = 0.890) excluding AD diagnoses in the first 5 years of the study. Similarly, Beydoun et al. (2024) used data from the NHANES and the Centers for Medicare and Medicaid Services (CMS-Medicare) from the United States in their longitudinal investigation that included HSV-2 and AD. Logistic regression modeling adjusted for covariates including age, sex, race/ethnicity, poverty income ratio, education, urban-rural area of residence, household size, marital status, nutritional factors and biomarkers, lifestyle, health-related factors, dentate status, and social support variables showed an adjusted logHR of 0.16 (SE = 0.19, *p*-value of 0.39), suggesting no association between HSV-2 and AD.

Further, despite meeting all our inclusion criteria, one study (Lindman et al., 2021) had a smaller sample that appeared to overlap with that of another study meeting our inclusion criteria (Lövheim et al., 2018), so we did not include it in the meta-analysis. However, its results mirrored those of our meta-analysis, finding no significant association between HSV-2 and AD (*p* = 0.45; Lindman et al., 2021). To further investigate this, we ran exploratory analyses replacing the results from Lövheim et al. (2018) with Lindman et al. (2021) to see how results would change. While the results shifted slightly, the conclusions did not change.

Although not meeting criteria for inclusion in our meta-analysis, several other studies warrant discussion when considering a possible association between dementia and HSV-2, with both mechanistic and epidemiological views. One such study examining some potential mechanistic relationships from a sample from the United States found no significant association between HSV-2 and AD (*HR*: 0.92, 95% CI: 0.79, 1.09) and even found HSV-2 to be protective for all-cause dementia (*HR*: 0.79, 95% CI: 0.75, 0.84) (Young-Xu et al., 2021). However, this study included patients in the HSV-2 group with a negative HSV test if treated with an HSV antiviral regimen, precluding inclusion in the present meta-analysis (Young-Xu et al., 2021). These investigators attributed this neuroprotective effect largely to the antiherpetic medication taken by many of the patients (Young-Xu et al., 2021). Another study found a significant unadjusted association between HSV-2 and mild cognitive impairment and dementia (*OR*: 2.12, 95% CI: 1.22, 3.69) (Shi et al., 2024). However, with adjustment, the association was no longer significant (*OR*: 1.71, 0.88, 3.33) (Shi et al., 2024). Corroborating these findings, a two-sample Mendelian Randomization study found no causal association between HSV-2 IgG and AD (inverse-variance weighted *OR*: 0.96, 95% CI: 0.79, 1.18) (Zhang et al., 2022). In a study that evaluated neuroimaging markers of dementia including brain volume, hippocampal volume, and white matter lesions, samples seropositive for HSV-2 showed no significant difference in neuroimaging outcomes (Green et al., 2023). To add to this, another study investigated some potential epidemiological relationships and examined postmortem AD brains for HSV-2, HHV-6, HSV-1, and CMV DNA compared to age-matched healthy controls (Lin et al., 2002). While samples positive for HSV-1 and HHV-6 show an increase in AD prevalence, HSV-2 cases did not. In a study of patients with schizophrenia, bipolar disorder, and healthy controls, HSV-2 seropositivity was not associated with any cognitive domain analyzed, though potential associations emerged when examining infectious disease burden, non-specific to HSV-2, specifically in working memory (Hamdani et al., 2017).

In contrast with reported findings not showing associations between HSV-2 and either dementia or cognitive function, several studies have found an association between HSV-2 and cognition or reduced cortical thickness. Using MRI to measure hippocampal volume, whole-brain cortical thickness, and an AD-related cortical thickness signature, Roberts et al. (2023) found that HSV-2 exposure was significantly associated with reduced whole-brain cortical thickness but not associated with hippocampal volume or the AD-related signature. This potential relationship was also seen through the results and correlations of HSV-2 and cognitive test outcomes. Significantly, Nimgaonkar et al. (2016) reported a decrease in cognitive performance with an increase in HSV-2 IgG antibody titers across all cognitive tests, including those measuring attention, executive function, memory, language, visuospatial function, and general cognition [using the Mini-Mental State Examination (MMSE)]. Longitudinally, HSV-2 IgG antibody levels brought a further decrease in memory, measured annually for 5 years. Similarly, Strandberg et al. (2003) found a significant association between HSV-2 IgG seropositivity and cognitive impairment, measured by the MMSE, in individuals with cardiovascular disease. Further, Deng et al. (2016) found that 23.8% of patients with vascular cognitive impairment, but not dementia, tested positive for HSV-2, compared to only 6.8% of controls (*p* < 0.001).

Overall, findings from studies evaluating associations between HSV-2 and dementia are mixed with findings showing no effect or even neuroprotective effects (Green et al., 2023; Hamdani et al., 2017; Lin et al., 2002; Shi et al., 2024; Young-Xu et al., 2021; Zhang et al., 2022), in contrast to other findings which show significant decline in cognitive function, decrease in cortical thickness, or increased incidence of dementia in groups seropositive for HSV-2 (Deng et al., 2016; Nimgaonkar et al., 2016; Roberts et al., 2023; Strandberg et al., 2003). These differences could be due to a variety of reasons such as heterogeneity among samples including differences in medical, sociodemographic, and geographical factors.

Exposure to other infectious diseases in addition to HSV-2 also could affect the association between HSV-2 and dementia. In this regard, several studies have examined associations between an infection burden and dementia, albeit with mixed results (Lin et al., 2002). However, previous work has suggested increased risk of dementia with two infectious diseases verses one (Sipilä et al., 2021). Furthermore, interactions between specific pathogens and the overall infection burden have been found to increase the risk of dementia (Beydoun et al., 2024). Several studies have shown that interactions between specific pathogens may increase the risk of dementia, including those between *Helicobacter pylori* and various periodontal pathogens (Beydoun et al., 2021), HSV-1 and HSV-2 (*OR*: 3.47, 95% CI: 2.30, 5.23; Araya et al., 2025), and unspecified HSV and varicella-zoster virus (Shin et al., 2024). Of note, one study found a significant association between CMV and HSV-1 when investigating the interaction between the two (*OR*: 5.662, 95% CI: 1.61, 19.97; Lövheim et al., 2018). A similar scenario could plausibly be the case with HSV-2, as it could be that it is the interaction between HSV-2 and other infectious pathogens that increases risk of dementia. Specifically, CMV may be a beneficial target of future interaction studies with HSV-2 given its established roles as a neurotropic virus and as a significant risk factor for dementia (Sanami et al., 2024). In investigating these interactions, it would be helpful to specifically look at additive vs. multiplicative interactions to elucidate the pathways by which these potential risk factors lead to dementia.

Time since infection could be an additional factor that requires consideration when evaluating associations between HSV-2 and dementia. In the case of infectious diseases and dementia, several studies have shown a decreased magnitude of the risk of dementia as time post-infection increases, whether that be hospital-treated infections, general common infections, or herpes viruses specifically (Drinkall et al., 2025; Janbek et al., 2023; Muzambi et al., 2021). If this is true of the association between HSV-2 and dementia, studies excluding recently diagnosed HSV-2 could possibly reduce the strength of the association between HSV-2 and dementia. Accordingly, more research reporting median time-to-event could further elucidate the temporal association between HSV-2 and dementia.

### 4.1 Limitations

Several additional factors require consideration when evaluating our findings. First, performing a meta-analysis with few studies is statistically challenging. It is difficult to get precise estimates of the heterogeneity variance, and there is limited data to explore the potential sources of heterogeneity, as with a meta-regression (Lin, 2018; von Hippel, 2015). Further, prediction intervals often become uninformative since they almost always cover the null simply due to the lack of studies. These challenges result in meta-analyses of few events being inherently underpowered. Ironically, a large benefit of meta-analysis is that power is increased, but when random-effects methods are used, there is often not an increase in power when fewer than five studies are included in the analysis (Jackson and Turner, 2017).

Second, meta-analyzing studies with rare events, such as cases of dementia, presents unique statistical challenges. Estimating the between-study heterogeneity is highly imprecise when events are rare. Consequently, the meta-analysis of HSV-2 and all-cause dementia is constrained by both the small number of available studies and the rarity of events, making precise and reliable estimation difficult. While a Bayesian approach could have been employed, such methods typically require informative priors to offer improvements over frequentist approaches when few studies are available (Bender et al., 2018). In this case, we lacked sufficient prior information to justify using informative priors, and employing uninformative priors would not have provided additional benefits.

Third, the beta-binomial model has primarily been studied in the context of randomized controlled trials (RCTs). One notable characteristic of this model is that it “breaks randomization” by not accounting for the pairing of treatment and control groups within individual studies. While previous research has shown that this does not significantly impact the beta-binomial model's performance with data from RCTs (Mathes and Kuss, 2018), our exposure data may be more sensitive to this break in randomization. Further research is needed to assess how this limitation affects the model's performance in non-randomized or observational data contexts.

An additional limitation concerns the potential for publication bias. While the small number of primary studies precluded formal assessments for publication bias, the results of the meta-analyses are susceptible to additional potentially null results from unpublished studies, which would further weaken the possible association we found between HSV-2 and all-cause dementia. As such, and in the context of the other limitations of this study, the HR results should be considered provisional and merely hypothesis generating.

Lastly, the present study focused on HSV-2 seropositivity as the primary exposure. In reality, seropositivity does not strictly signify neurovirulence, meaning that it may be possible that the exposed samples in these primary studies had HSV-2 IgG seropositivity without HSV-2 in the brain. However, HSV-2 IgG was robustly associated with whole-brain cortical thickness, representing an association between seropositivity and neuropathological changes (Roberts et al., 2023). Further, for the purpose of comparing similar methods within the present meta-analysis, we included studies only investigating IgG, as opposed to any HSV-2 exposure, although investigation of HSV-2 directly in the brain using polymerase chain reactions showed varied results (Warren-Gash et al., 2019).

In conclusion and in the context of the limitations associated with this meta-analysis, particularly the low number of primary studies, the present findings indicate a possible association between HSV-2 and dementia. Further work is warranted to better explore this association, with a particular focus placed on dementia subtype, medical and sociodemographic factors, timing of the HSV-2 infection, and interactions between HSV-2 and other infectious diseases. Better characterization of the relationship between HSV-2 and dementia is crucial, considering findings showing that antiherpetic treatment for HSV may lower risk of incident dementia (Readhead et al., 2018; Tzeng et al., 2018), a finding with clinical and public-health implications if antiherpetic treatment lowers dementia risk in patients seropositive for HSV-2.

## Data Availability

The original contributions presented in the study are included in the article/Supplementary material, further inquiries can be directed to the corresponding author.
