# Supplementary material for: Herpes simplex virus 2 and dementia risk: a systematic review and meta-analysis
Source: Front Dement. 2026 Mar 24;5:1737068. doi: 10.3389/frdem.2026.1737068 (PMC13053239; doi:10.3389/frdem.2026.1737068)
Supplement: Supplementary file 1 [file Data_Sheet_1.docx]

Supplementary Material

# Full Search Terms Used

("HSV 2" OR "Herpes Simplex Virus 2" OR "Herpes Simplex Virus II" OR "HSV2" OR "HSVII" OR "HSV-II" OR "Herpes Simplex Virus Type 2" OR "Herpesvirus 2 (alpha), Human" OR "HHV-2" OR "HSV-2" OR "Human Herpesvirus 2") AND (("Lewy Body" OR "Diffuse Lewy Body Disease" OR "Dementia, Lewy Body" OR "Cortical Lewy Body Disease" OR "Lewy Body Disease, Cortical" OR "Lewy Body Type Senile Dementia" OR "Lewy Body Disease, Diffuse" OR "Lewy Body Dementia" OR "Frontotemporal dementia" OR "Dementias, Frontotemporal" OR "Frontotemporal Dementias" OR "Frontotemporal Lobe Dementia (FLDEM)" OR "Dementia, Frontotemporal Lobe (FLDEM)" OR "Dementias, Frontotemporal Lobe (FLDEM)" OR "Frontotemporal Lobe Dementias (FLDEM)" OR "Frontotemporal Dementia with Parkinsonism" OR "Dementia, Frontotemporal, with Parkinsonism" OR "Multiple System Tauopathy with Presenile Dementia" OR "Frontotemporal Dementia, Ubiquitin-Positive" OR "Dementias, Ubiquitin-Positive Frontotemporal" OR "Dementia, Ubiquitin-Positive Frontotemporal" OR "Frontotemporal Dementias, Ubiquitin-Positive" OR "Frontotemporal Dementia, Ubiquitin Positive" OR "Ubiquitin-Positive Frontotemporal Dementia" OR "Ubiquitin-Positive Frontotemporal Dementias" OR "Dementia, Hereditary Dysphasic Disinhibition" OR "Dementia, Frontotemporal" OR "Frontotemporal Lobe Dementia" OR "Dementia, Frontotemporal Lobe" OR "Dementias, Frontotemporal Lobe" OR "Frontotemporal Lobe Dementias" OR "Lobe Dementia, Frontotemporal" OR "Lobe Dementias, Frontotemporal" OR "Wilhelmsen-Lynch Disease" OR "Diseases, Wilhelmsen-Lynch" OR "Disease, Wilhelmsen-Lynch" OR "Wilhelmsen Lynch Disease" OR "Wilhelmsen-Lynch Diseases" OR "Frontotemporal Lobar Degeneration With Ubiquitin-Positive Inclusions" OR "Frontotemporal Lobar Degeneration With Ubiquitin Positive Inclusions" OR "Disinhibition-Dementia-Parkinsonism-Amyotrophy Complex" OR "Complex, Disinhibition-Dementia-Parkinsonism-Amyotrophy" OR "Complices, Disinhibition-Dementia-Parkinsonism-Amyotrophy" OR "Disinhibition Dementia Parkinsonism Amyotrophy Complex" OR "Disinhibition-Dementia-Parkinsonism-Amyotrophy Complices" OR "Frontotemporal Dementia with Parkinsonism-17" OR "Frontotemporal Dementia with Parkinsonism 17" OR "Disinhibition-Dementia-Parkinsonism-Amytrophy Complex" OR "Complex, Disinhibition-Dementia-Parkinsonism-Amytrophy" OR "Complices, Disinhibition-Dementia-Parkinsonism-Amytrophy" OR "Disinhibition Dementia Parkinsonism Amytrophy Complex" OR "Disinhibition-Dementia-Parkinsonism-Amytrophy Complices" OR "Familial Pick's Disease" OR "Disease, Familial Pick's" OR "Diseases, Familial Pick's" OR "Familial Pick Disease" OR "Familial Picks Disease" OR "Familial Pick's Diseases" OR "Pick's Disease, Familial" OR "Pick's Diseases, Familial" OR "Hereditary Dysphasic Disinhibition Dementia" OR "FTDP-17" OR "DDPAC" OR "GRN-Related Frontotemporal Dementia" OR "Dementia, GRN-Related Frontotemporal" OR "Dementias, GRN-Related Frontotemporal" OR "Frontotemporal Dementia, GRN-Related" OR "Frontotemporal Dementias, GRN-Related" OR "GRN Related Frontotemporal Dementia" OR "GRN-Related Frontotemporal Dementias" OR "FTD-GRN" OR "FTD-PGRN" OR "FTLD-17 GRN" OR "FTLD with TDP-43 Pathology" OR "FTLD with TDP 43 Pathology" OR "FTLD-TDP" OR "HDDD1" OR "HDDD2" OR "Semantic Dementia" OR "Dementia, Semantic" OR "Dementias, Semantic" OR "Semantic Dementias" OR "Dementia" OR "Dementias" OR "Amentia" OR "Amentias" OR "Senile Paranoid Dementia" OR "Dementias, Senile Paranoid" OR "Paranoid Dementia, Senile" OR "Paranoid Dementias, Senile" OR "Senile Paranoid Dementias" OR "Familial Dementia" OR "Dementia, Familial" OR "Dementias, Familial" OR "Familial Dementias" OR "AD" OR "Alzheimer Syndrome" OR "Alzheimer-Type Dementia (ATD)" OR "Alzheimer Type Dementia (ATD)" OR "Dementia, Alzheimer-Type (ATD)" OR "Alzheimer's Diseases" OR "Alzheimer Diseases" OR "Alzheimers Diseases" OR "Alzheimer Dementia" OR "Alzheimer Dementias" OR "Dementia, Alzheimer" OR "Alzheimer's Disease" OR "Dementia, Senile" OR "Senile Dementia" OR "Dementia, Alzheimer Type" OR "Alzheimer Type Dementia" OR "Senile Dementia, Alzheimer Type" OR "Alzheimer Type Senile Dementia" OR "Primary Senile Degenerative Dementia" OR "Alzheimer Sclerosis" OR "Sclerosis, Alzheimer" OR "Dementia, Primary Senile Degenerative" OR "Dementia, Presenile" OR "Presenile Dementia" OR "Acute Confusional Senile Dementia" OR "Senile Dementia, Acute Confusional" OR "Alzheimer Disease, Early Onset" OR "Early Onset Alzheimer Disease" OR "Presenile Alzheimer Dementia" OR "Alzheimer Disease, Late Onset" OR "Late Onset Alzheimer Disease" OR "Alzheimer's Disease, Focal Onset" OR "Focal Onset Alzheimer's Disease" OR "Familial Alzheimer Disease (FAD)" OR "Alzheimer Disease, Familial (FAD)" OR "Familial Alzheimer Diseases (FAD)" OR "Cognitive Dysfunctions" OR "Dysfunction, Cognitive" OR "Dysfunctions, Cognitive" OR "Cognitive Disorder" OR "Cognitive Disorders" OR "Disorder, Cognitive" OR "Disorders, Cognitive" OR "Cognitive Impairments" OR "Cognitive Impairment" OR "Impairment, Cognitive" OR "Impairments, Cognitive" OR "Mild Cognitive Impairment" OR "Cognitive Impairment, Mild" OR "Cognitive Impairments, Mild" OR "Impairment, Mild Cognitive" OR "Impairments, Mild Cognitive" OR "Mild Cognitive Impairments" OR "Cognitive Decline" OR "Cognitive Declines" OR "Decline, Cognitive" OR "Declines, Cognitive" OR "Mental Deterioration" OR "Deterioration, Mental" OR "Deteriorations, Mental" OR "Mental Deteriorations")[Title/Abstract])
